# Erythropoietin Improves Atrophy, Bleeding and Cognition in the Newborn Intraventricular Hemorrhage

**DOI:** 10.3389/fcell.2020.571258

**Published:** 2020-09-16

**Authors:** Carmen Hierro-Bujalance, Carmen Infante-Garcia, Daniel Sanchez-Sotano, Angel del Marco, Ana Casado-Revuelta, Carmen Maria Mengual-Gonzalez, Carmen Lucena-Porras, Marcos Bernal-Martin, Isabel Benavente-Fernandez, Simon Lubian-Lopez, Monica Garcia-Alloza

**Affiliations:** ^1^Division of Physiology, School of Medicine, Universidadde Cádiz, Cádiz, Spain; ^2^Instituto de Investigacion e Innovacion en Ciencias Biomedicas de la Provincia de Cádiz (INiBICA), Cádiz, Spain; ^3^Division of Paediatrics, Section of Neonatology, Hospital Universitario Puerta del Mar, Cádiz, Spain

**Keywords:** brain atrophy, neuronal loss, erythropoietin, gelsolin, germinal matrix-intraventricular hemorrhage, preterm infant

## Abstract

The germinal matrix-intraventricular hemorrhage (GM-IVH) is one of the most devastating complications of prematurity. The short- and long-term neurodevelopmental consequences after severe GM-IVH are a major concern for neonatologists. These kids are at high risk of psychomotor alterations and cerebral palsy; however, therapeutic approaches are limited. Erythropoietin (EPO) has been previously used to treat several central nervous system complications due to its role in angiogenesis, neurogenesis and as growth factor. In addition, EPO is regularly used to reduce the number of transfusions in the preterm infant. Moreover, EPO crosses the blood-brain barrier and EPO receptors are expressed in the human brain throughout development. To analyze the role of EPO in the GM-IVH, we have administered intraventricular collagenase (Col) to P7 mice, as a model of GM-IVH of the preterm infant. After EPO treatment, we have characterized our animals in the short (14 days) and the long (70 days) term. In our hands, EPO treatment significantly limited brain atrophy and ventricle enlargement. EPO also restored neuronal density and ameliorated dendritic spine loss. Likewise, inflammation and small vessel bleeding were also reduced, resulting in the preservation of learning and memory abilities. Moreover, plasma gelsolin levels, as a feasible peripheral marker of GM-IVH-induced damage, recovered after EPO treatment. Altogether, our data support the positive effect of EPO treatment in our preclinical model of GM-IVH, both in the short and the long term.

## Introduction

Every year there are over 15 million preterm births and ∼7% of these kids suffer long term neurodevelopmental impairment (Maxwell et al., 2017). The germinal matrix-intraventricular hemorrhage (GM-IVH) is one of the most common complications observed in the preterm infant (PTI) (Tan et al., 2018). While the incidence of GM-IVH has declined since the 1990s, due to improvements in neonatal care, the increased survival of extremely PTI has also contributed to maintain the absolute number of GM-IVH very high (Mukerji et al., 2015). GM-IVH in premature infants arises from the germinal matrix (GM), which is a complex vascularized layer rich in immature vessels that surrounds the lateral ventricles, corresponding with the subventricular zone (SVZ), from where neurons and glial cells rise during fetal development. GM microvasculature is extremely fragile due to an abundance of angiogenic blood vessels that exhibit paucity of pericytes, immaturity of basal lamina and deficiency of glial fibrillary acidic protein in the ensheathing astrocytes endfeet (Ballabh, 2010). Therefore, PTI with very low weight birth are more susceptible to local hemorrhage (Perlman, 2009). The short- and long-term neurodevelopmental complications in the PTI with severe or even mild GM-IVH are a major concern, since these kids are at high risk of neurosensory impairment, developmental delay, cerebral palsy, deafness, psychomotor alterations or cerebral palsy (Ballabh, 2010; Mukerji et al., 2015; Benavente-Fernandez et al., 2018).

Therapeutic approaches focus on enhancing the stability of GM vasculature and regulating the cerebral blood flow (Ballabh, 2010). Following this idea, erythropoietin (EPO) promotes the survival, proliferation and differentiation of erythrocyte progenitors in bone marrow (Lombardero et al., 2011) and is regularly used in PTI to reduce the number of transfusions. EPO crosses the blood-brain barrier in a dose-dependent manner and EPO receptors are expressed in the human brain throughout development (Kollensperger et al., 2011). Previous studies have reported that EPO can play a potential role in angiogenesis and neurogenesis (Lombardero et al., 2011; Juul and Pet, 2015). Thus, EPO has been used to treat infants with hypoxic-ischemic encephalopathy (Razak and Hussain, 2019). EPO treatment to PTI has shown a significant decrease of white matter injury and it might improve neurodevelopmental outcome of extremely PTI (Neubauer et al., 2010), although other studies failed to detect significant benefits, as recent PENUT study has recently reported (Juul et al., 2020). At present, EpoRepair (Erythropoietin for the Repair of Cerebral Injury in Very Preterm Infants) trial (Ruegger et al., 2015) outcomes are also awaited soon. Therefore, the role of EPO in GM-IVH of the PTI remains unclear. To deepen at this level, we have induced a GM-IVH in mice by intraventricular administration of collagenase (Col). After EPO treatment, brain atrophy and ventricular dilatation were significantly reduced. Spine and neuronal loss were limited, and overspread small vessel damage and inflammation were reduced, resulting in the preservation of learning and memory abilities. Interestingly, plasma gelsolin levels, as a feasible peripheral marker of GM-IVH-induced damage, previously observed in other brain disorders (Xu et al., 2012; Peng et al., 2015; Benavente-Fernandez et al., 2018), recovered after EPO administration.

## Materials and Methods

### Animals and Treatments

Germinal matrix-intraventricular hemorrhage was induced to male and female CD1 mice at P7 by intracerebroventricular (icv) infusion of Col VII-S (batch number: SLBG8830V Sigma, St Louis, MO) (Segado-Arenas et al., 2018). Mice were anesthetized with isoflurane and placed in a stereotaxic frame (David-Kopf, California, United States). 0.3 IU of Col in 1 μl of TESCA 50 mM (TES buffer, Sigma, ref. T1375, St. Louis, MO, United States) and calcium chloride anhydrous 0.36 mM (Sigma, ref. C1016, St. Louis, MO, United States), pH7.4 and 37°C, were injected with Hamilton syringe in the right ventricle at 0.2 μl/min (+ 5 more minutes to avoid the retraction of the liquid). Sham animals received 1 μl of TESCA. Control mice received no injection. EPO treatment (10.000 or 20.000 IU) (EPO10 and EPO20) was ip injected on 3 consecutive days: immediately after completing the lesions, as well as 24 and 48 h later (Kaindl et al., 2008; Fan et al., 2011; Zhang et al., 2020). A set of mice (10–12/group) were sacrificed at P14 to analyze the early effects of EPO and a second set (10–12/group) were aged up to P70 to assess the long-term effects of EPO treatment. Two weeks before sacrifice mice underwent behavioral assessment. Animals that died before the experiments were completed were not included in subsequent analyses. All animals were maintained in the Animal Facility of the University of Cadiz under 12 h light/dark cycles and controlled temperature (21 ± 2°C) with *ad libitum* access to food and water. All experimental procedures were approved by the University of Cadiz Bioethical Committee (Guidelines for Care and Use of Experimental Animals, European Commission Directive 2010/63/UE and Spanish Royal Decree 53/2013).

### Rotarod

Rotarod (Ugo Basile Srl; Varese, Italia) was used to evaluate motor skills as described (Segado-Arenas et al., 2018). Mice were placed in the cylinder 4 min at 4 rpm for habituation purposes. During the test speed was progressively increased from 4 to 60 rpm, in 1 min, and the time spent on the rotarod was recorded.

### Morris Water Maze

A round pool (0.95 m in diameter) was filled with water (21 ± 1°C) and four equal virtual quadrants were indicated with geometric cues located in the walls. Experiments were run as previously described (Infante-Garcia et al., 2017). Briefly, the scape platform was invisible, 2–3 cm below the surface. The acquisition started starting 12 days before sacrifice and consisted of 4 trials/day for 4 days with the submerged platform in the virtual quadrant number 2. Time limit to locate the platform was 60 s/trial with an intertrial interval of 10 min. When the animal did not find the platform it was manually placed on it for 10 s. Retention 1 commenced 24 h after the finalization of the acquisition phase, and retention 2 was run 72 h after the end of the acquisition phase. Retention phases consisted in a single trial with the platform removed. Time required to locate the platform (acquisition), percentage of time spent in quadrant 2 (retention) and swimming velocity were analyzed using Smart software, Panlab (Spain).

### Motor Activity and New Object Discrimination Task

Spontaneous motor activity was analyzed by measuring the distance traveled by mice for 30 min in a rectangular box (22 × 44 × 40 cm), as described (Infante-Garcia et al., 2016). The next day mice were placed in the same box and exposed to two objects (not used afterward), for habituation purposes. On the third day mice were exposed to two sample trials and a test trial. On the first sample trial, mice were placed into the center of the box containing 3 copies of an object arranged in a triangle-shaped spatial configuration. Mice explored for 5 min and after 30 min delay they received a second sample trial with 4 novel objects, arranged in a quadratic-shaped spatial configuration, for another 5 min. Test trial was performed after 30 min delay and mice were exposed to 2 copies of the object from sample trial 2 (“recent” objects) placed at the same position, and 2 copies of the object from sample trial 1 (“familiar” objects) placed one of them at the same position (“familiar non-displaced” object) and the another in a new position (“familiar displaced” object). “What,” “where” and “when” integrated episodic memories were analyzed as described (Infante-Garcia et al., 2016).

### Cresyl Violet Staining

We analyzed brain morphology in 6 sections 1 mm apart (from 1.5 to −3.5 mm from Bregma). Sections were dehydrated in 70% ethanol for 15 min. They were stained with cresyl violet (Sigma, St. Louis, MO, United States) solution (0.5% w/v) for 10 min, as previously described (Ramos-Rodriguez et al., 2017). Sections were then fixed sequentially in 0.25% acetic acid, 100% ethanol and xylene for 2 min. Sections were mounted with DPX (Sigma, St. Louis, MO, United States). Analysis was conducted blind to the experimental conditions. Images were acquired using an optical Olympus Bx60 microscope (Japan) with an attached Olympus DP71 camera and Cell F software (Olympus, Hamburg, Germany). Cortex and lateral ventricle sizes were measured using Adobe Photoshop and Image J software.

### NeuN and Microglia Immunostaining

NeuN and microglia immunohistochemistry was performed as described (Infante-Garcia et al., 2015). Anti-NeuN (Chemicon, CA, United States) (1:200) and anti-Iba1 (Wako, Osaka, Japan) (1:1000) were used as primary antibodies for neuron and microglia assessment and incubated overnight at 4°C. Alexa Fluor 594 or Alexa Fluor 488 (1:1000), respectively, were used as secondary antibodies. DAPI 1mg/ml (Sigma) (1:3000) counterstain was used after NeuN immunohistochemistry and the percentage of NeuN-positive cells (normalized by total cells stained with DAPI) was quantified in the cortex and the SVZ using Image J software (Ramos-Rodriguez et al., 2016). Analysis was conducted blind to the experimental conditions. Number of microglia cells, individual microglia size and burden were quantified in the cortex and the SVZ using Image J software (Infante-Garcia et al., 2016).

### Golgi-Cox Staining

Neuron architecture was further analyzed by Golgi-Cox staining, using Rapid Golgi Stain Kit (FD Neurotechnologies, United States. Ref: PK401) following manufacturer’s indications with minor modifications. Brains were maintained in the kit solutions for 3 weeks, and in 3% agarose liquid solution afterward. Blocks were cut into 200 μm on a vibratome (Ted Pella, Inc., California, United States) Sections were stained as indicated by manufacturer. Sections were dehydrated with ethanol and mounted with DPX (Sigma, United States). Neurons were photographed with Olympus U-RFLT microscope (Olympus, Japan), using MMI Cell Tools software. Neuronal complexity was analyzed by sholl analysis in 10 μm concentric circles from neuronal soma. Analysis was conducted blind to the experimental conditions. Number of crossings every 10 μm were quantified. Spine density was calculated (spines/10 μm) (Infante-Garcia et al., 2015). Ratios of curvature were calculated by dividing the end-to-end distance of a dendrite segment by the total length between the two segment ends using Image J software (Ramos-Rodriguez et al., 2017).

### Prussian Blue Staining

Consecutive sections to those used for cresyl violet staining were used for Prussian blue iron staining and neutral red counterstaining to analyze the presence of hemorrhages (Infante-Garcia et al., 2015). Images were acquired using an optical Olympus Bx60 microscope (Japan) with an attached Olympus DP71 camera and Cell F software (Olympus, Hamburg, Germany). Analysis was conducted blind to the experimental conditions. Hemorrhage burden (% of area covered by hemorrhages) was analyzed in the SVZ (up to 20 μm from the lateral ventricles), the cortex and the hippocampus using ImageJ software.

### Western Blot for Tau and Akt

We quantified phopho-tau, total tau, phospo-Akt and total Akt levels in the striatum (including the SVZ), cortex and hippocampus from all animals under study. Samples were prepared as previously described (Infante-Garcia et al., 2016). Briefly, tissue was homogenized in lysis buffer (Cell Signaling, United States) with a protease/phosphatase inhibitor cocktail (Sigma, United States). After centrifugation for 5 min, at 15000 *g* and 4 °C Bradford (Bio-Rad, Germany) protein assay was used for protein concentration in supernatants. Proteins were separated on 10% acrylamide-bisacrylamide gels, followed by electrophoretic transfer to PVDF membranes (Schleicher & Schuell, Keene, NH). Antibodies used included: phospho-tau clone AT8 (1:1000, Fisher Scientific, MA, United States), phospho-Akt (Ser473) (1:1000) (Cell signaling, United States), anti-total Akt antibody (1:2000) (cell signaling, United States) and anti-total tau antibody (1:1000) (DAKO, Denmark). Optical density was semi-quantified after normalizing to β-actin (1:2,500,000) (Sigma, United States), using ImageJ software. Data were represented as percentage of Control values.

### Matrix Metalloproteinase 9 and Gelsolin Plasma Determinations

Plasma matrix metalloproteinase 9 (MMP9) and gelsolin levels were measured, as feasible markers of central damage, in the short (P14) and the long (P70) term. We used ELISA kits for MMP9 (R&D System Corp, MN, United States) and gelsolin (Cusabio Biotech Co., Wuhan, Hubei Province, China), following manufacturer’s indications as previously described (Segado-Arenas et al., 2018).

### Statistical Analysis

One-way ANOVA, followed by Tuckey b test or Tamhane tests as required, were used. No statistical differences were detected between Sham and Control groups, therefore all animals were combined in a single Control group. Two-way ANOVA was used to analyze the MWM test (groupXday) and neurites architecture (groupXradius). SPSS v.15 software was used for all statistical analysis.

## Results

### EPO Significantly Restores Cognitive Impairment After Inducing a GM-IVH

**FIGURE 1:**
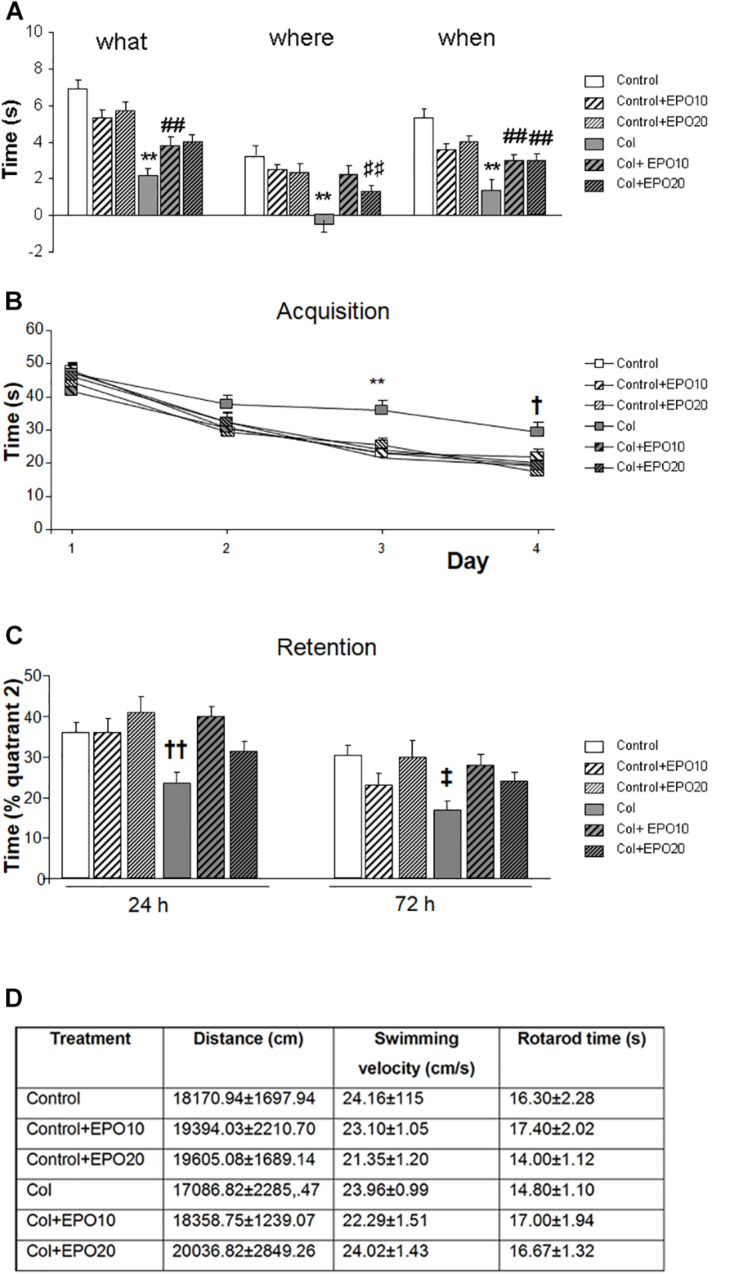
EPO treatment significantly reduces GM-IVH cognitive impairment at P70. **(A)** Episodic memory is significantly impaired at P70 after inducing a GM-IVH, and EPO10 and EPO20 treatment counterbalances this limitation for “what” (**p < 0.01 vs. rest of the groups, ##*p* < 0.01 vs. Control and Control + EPO20), “where” (**p < 0.01 vs. rest of the groups, ⊺⊺*p* < 0.01 vs. Control) and “when” (**p < 0.01 vs. rest of the groups, ##*p* < 0.01 vs. Control and Control + EPO20) paradigms. **(B)** Spatial memory was also impaired after Col lesions, when analyzed in the acquisition phase of the Morris water maze, while EPO treatments significantly reversed this situation on individual days (day 1: *p* = 0.496, day 2: *p* = 0.437, day 3: **p = 0.004 vs. rest of the groups, day 4: †*p* = 0.025 vs. Control, Control + EPO10, Control + EPO20 and Col + EPO20). **(C)** A similar profile was observed in the retention phase and EPO10 and EPO20 treatment significantly improved the time spent where the platform used to be located, 24 (††*p* = 0.002 vs. Control, Control + EPO10, Control + EPO20 and Col + EPO10) and 72 h (‡*p* = 0.025 vs. Control and Col + EPO20) after completing the acquisition phase. **(D)** Motor skills were not affected in rotarod (*p* = 0.962), spontaneous motor activity (*p* = 0.917) and swimming velocity (*p* = 0.566).

The overall compromise in GM-IVH mice for “what,” “where” and “when” paradigms in the new object discrimination test, was significant improvement after EPO treatment (“what” [*F*_(__5_,_225__)_ = 11.30, ^∗∗^*p* < 0.01 vs. rest of the groups, ##*p* < 0.01 vs. Control and Control + EP20], “where” [*F*_(__5_,_249__)_ = 8.36, ^∗∗^*p* < 0.01 vs. rest of the groups, ##*p* < 0.01 vs. Control], “when” [*F*_(__5_,_258__)_ = 10.95, ^∗∗^*p* < 0.01 vs. rest of the groups, ##*p* < 0.01 vs. Control and Control + EP20]) (Figure 1A). Similar results were detected in the Morris water maze test. We did not detect a significant groupXday effect [*F*_(__15_,_1335__)_ = 0.859, *p* = 0.611], although further daily assessment revealed differences on individual acquisition days (day 1 [*F*_(5,371)_ = 0.878, *p* = 0.496], day 2 [*F*_(5,344)_ = 0.969, *p* = 0.437], day 3 [*F*_(5,351)_ = 3.498, ^∗∗^*p* = 0.004 vs. rest of the groups], day 4 [*F*_(5,358)_ = 2.59, †*p* = 0.025 vs. Control, Control + EPO10, Control + EPO20 and Col + EPO20]) (Figure 1B). Also in the retention phase, EPO10 and EPO20 significantly improved Col-induced impairment (24 h [*F*_(5,85)_ = 4.29, ††*p* = 0.002 vs. Control, Control + EPO10, Control + EPO20 and Col + EPO10]; 72 h [*F*_(5,89)_ = 2.75, ‡*p* = 0.025 vs. Control and Col + EPO20]) (Figure 1C). Time in rotarod ([*F*_(5,57)_ = 0.611, *p* = 0.962]), spontaneous motor activity ([*F*_(5,85)_ = 0.289, *p* = 0.917]) and swimming velocity ([*F*_(5,89__)_ = 0.781, *p* = 0.566]) (Figure 1D) were not affected, suggesting that alterations in learning and memory were motor-independent. However, we cannot exclude that larger lesions may reproduce motor-related alterations, as commonly observed in patients (Hinojosa-Rodríguez et al., 2017).

### EPO Treatment Reduces Brain Atrophy and Neuronal Alterations After Inducing a GM-IVH

**FIGURE 2:**
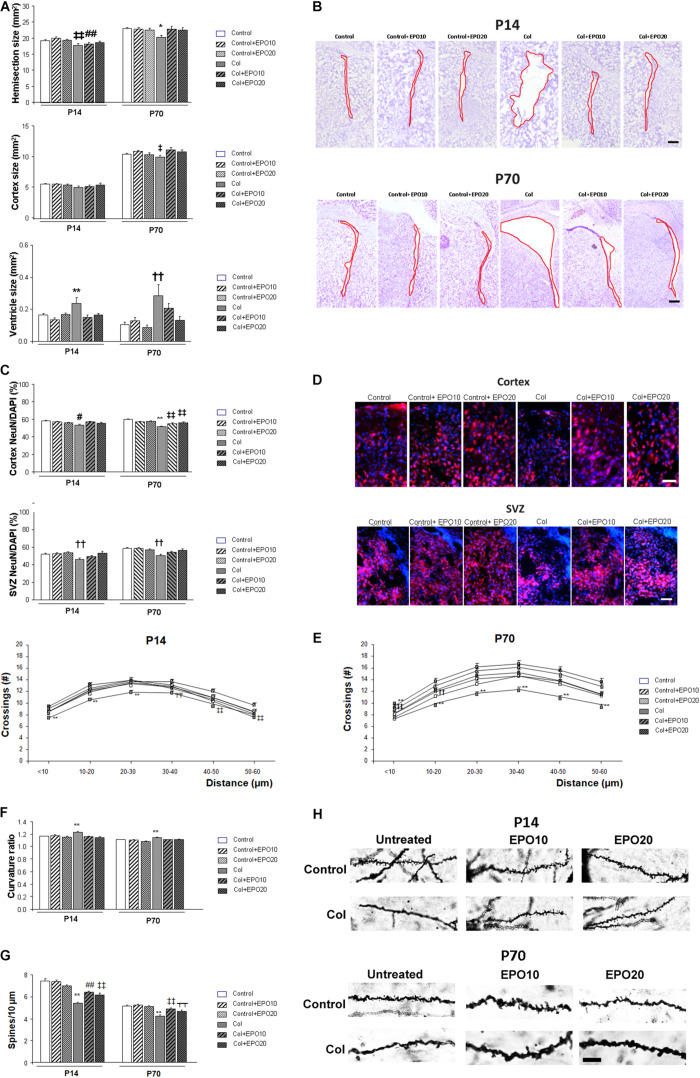
EPO treatment limits brain atrophy and neuronal alterations after induction of a GM-IVH with Col. **(A)** Ipsilateral hemisection size was reduced in P14 and P70 mice while EPO treatment limited this effect (P14: ‡‡*p* = 0.003 vs. Control, Control + EPO10 and Control + EPO20, ##*p* = 0.003 vs. Control + EPO10; P70: *p = 0.017 vs. rest of the groups). Differences observed in the cortex did not reach statistical significance at P14 (*p* = 0.358), although at P70 cortical compromise was ameliorated by EPO treatment (‡*p* = 0.014 vs. Control and Col + EPO10). Significant ventricle enlargement was detected at P14 and EPO treatment reversed this effect (**p = 0.001 vs. rest of the groups) and a similar profile was observed at P70 (††*p* = 0.001 vs. Control, Control + EPO10, Control + EPO20, Col + EPO20). **(B)** Illustrative example of cresyl violet staining showing the ipsilateral ventricle enlargement at P14 and P70, after inducing a GM-IVH, while ventricle size is maintained in mice treated with EPO. Red line borders the ventricle. Scale bar = 250 μm. **(C)** At P14, NeuN/DAPI ratio was reduced in the ipsilateral cortex and EPO treatment improved this situation (#*p* = 0.034 vs. Control, Control + EPO10 and Col + EPO10). A similar profile was observed at P70 (**p < 0.001 vs. rest of the groups, ‡‡*p* < 0.01 vs. Control, Control + EPO10 and Control + EPO20). The SVZ was also affected after Col lesions and EPO treatment significantly improved the NeuN/DAPI ratio both at P14 and P70 (P14: ††*p* = 0.002 vs. Control, Control + EPO10, Control + EPO20 and Col + EPO20; P70: ††*p* = 0.002 vs. Control, Control + EPO10, Control + EPO20 and Col + EPO20; P70). **(D)** Illustrative example of NeuN (red)/DAPI (blue) staining in the ipsilateral cortex and SVZ from mice with GM-IVH induced by Col, where a reduction in NeuN^+^cells can be observed, while more NeuN^+^cells are detected after EPO10 and EPO20 treatment. Scale 50 μm. **(E)** Neuronal complexity was affected by Col lesions and EPO treatment significantly improved this effect at P14 (**p < 0.01 vs. rest of the groups; ‡*p* = 0.017 vs. Cont + EPO20; ‡‡*p* = 0.003 vs. Cont + EPO20; T̄T̄*p* < 0.01 vs. rest Control and Cont + EPO20) or P70 (**p < 0.01 vs. rest of the groups, ‡‡*p* < 0.01 vs. Control, Control + EPO20; ††*p* < 0.01 vs. Control + EPO20, Col + EPO10 and Col + EPO20). **(F)** Neuron curvature ratio was also impaired after Col lesions and EPO treatment significantly ameliorated this situation both at P14 (**p < 0.01 vs. rest of the groups) and P70 (**p < 0.01 vs. rest of the groups). **(G)** Spine density compromise was also limited after EPO treatment in the ipsilateral hemispheres at P14 (**p < 0.01 vs. rest of the groups, ‡‡*p* < 0.01 vs. Control, Control + EPO10 and Control + EPO20, ##*p* < 0.01 vs. Control and Control + EPO10) and at P70 (**p < 0.01 vs. rest of the groups, ‡‡*p* < 0.01 vs. Control, Control + EPO10 and Control + EPO20, ⊺⊺*p* < 0.01 vs. Control + EPO10). **(H)** Illustrative example of spines labeled by Golgi-Cox staining. A reduction in spine density is observed in mice after lesions with Col and this effect is limited by EPO10 and EPO20 treatment at P14 and P70. Scale = 12.5 μm.

One week after Col lesions, the size of the ipsilateral hemispheres was reduced. EPO10 partially limited this effect, that was completely reversed by EPO20 treatment [*F*_(5,222__)_ = 3.775, ‡‡*p* = 0.003 vs. Control, Control + EPO10 and Control + EPO20, ##*p* = 0.003 vs. Control + 10]. The effect was still present at P70 [*F*_(__5,__174__)_ = 2.83, ^∗^*p* = 0.017 vs. rest of the groups] (Figure 2A). Reduced cortical size was slightly improved by EPO treatment, although differences did not reach statistical significance in the short term (P14) [*F*_(__5,__222__)_ = 1.107, *p* = 0.358]. In the long term (P70) EPO treatment improved cortical atrophy [*F*_(__5,__175__)_ = 2.95, ‡*p* = 0.014 vs. Control and Col + EPO10] (Figure 2A). Also, EPO treatment completely reversed ventricle enlargement after Col lesions at P14 [*F*_(__5,__104__)_ = 4.48, ^∗∗^*p* = 0.001 vs. rest of the groups] (Figures 2A,B). A similar profile was detected with EPO20 at P70 [*F*_(__5,__146__)_ = 4.21, ††*p* = 0.001 vs. Control, Control + EPO10, Control + EPO20, Col + EPO20] (Figure 2A). No statistical differences were observed when EPO10 and EPO20 treatments were compared, and even though a slightly higher improvement was observed in the cortex from EPO10-treated animals at P70, an overall better response was observed after EPO20 treatments in all the other paradigms under study, including cortical size at P14, as well as hemisection and ventricle sizes at P14 and P70.

At P14 NeuN/DAPI ratio was reduced in the ipsilateral cortex after Col lesions, and EPO treatment improved this situation [*F*_(__5,__3428__)_ = 2.41, #*p* = 0.034 vs. Control, Control + EPO10 and Col + EPO10] (Figures 2C,D). A similar outcome was observed at P70 [*F*_(__5,__2996__)_ = 14.35, ^∗∗^*p* < 0.001 vs. rest of the groups, ‡‡*p* < 0.01 vs. Control, Control + EPO10 and Control + EPO20] (Figures 2C,D). EPO20 treatment also improved NeuN/DAPI compromise in the SVZ at P14 [*F*_(__5,__987__)_ = 3.82, ††*p* = 0.002 vs. Control, Control + EPO10, Control + EPO20 and Col + EPO20] and P70 [*F*_(__5,__977__)_ = 4.73, ††*p* = 0.002 ‡vs. Control, Control + EPO10, Control + EPO20 and Col + EPO20] (Figures 2C,D).

Neuronal architecture was also altered after GM-IVH. Analysis of neurites intersections by sholl analysis after Golgi-Cox staining revealed a groupXradius distance effect by 2 way ANOVA in the ipsilateral hemisphere at P14 [*F*_(__25,__3997__)_ = 1.54, *p* = 0.041]. Further assessment of individual distances revealed and overall improvement in mice treated with EPO10 and EPO20 (<10 μm [*F*_(__5,__670__)_ = 7.59, ^∗∗^*p* < 0.01 vs. rest of the groups]; 10–20 μm [*F*_(__5,__669__)_ = 7.28, ^∗∗^*p* < 0.01 vs. rest of the groups]; 20–30 μm [*F*_(__5,__658__)_ = 5.08, ^∗∗^*p* < 0.01 vs. rest of the groups]; 30–40 μm [*F*_(__5,__656__)_ = 6.60, T̄T̄*p* < 0.01 vs. Control and Cont + EPO20]; 40–50 μm [*F*_(__5,__671__)_ = 3.60, ‡‡*p* < 0.01 vs. Cont + EPO20]; 50–60μm [*F*_(__5,__673__)_ = 3.87, ‡‡*p* = 0.002 vs. Cont + EPO20] (Figure 2E). At P70, we did not detect a significant groupXradius effect by 2 way ANOVA [*F*_(__25,__3779__)_ = 0.964, *p* = 0.514]. Assessment of individual distances revealed that EPO treatment limited neuronal simplification (<10 μm [*F*_(__5,__456__)_ = 11.10, ^∗∗^*p* < 0.01 vs. rest of the groups, ‡‡*p* < 0.01 vs. Control, Control + EPO20]; 10–20 μm [*F(*_5_,_668_) = 10.48, ^∗∗^*p* < 0.01 vs. rest of the groups; ††*p* < 0.01 vs. Control + EPO20, Col + EPO10 and Col + EPO20]; 20–30 μm [*F*_(__5,__655__)_ = 8.77, ^∗∗^*p* < 0.01 vs. rest of the groups]; 30–40 μm [*F*_(__5_,_662__)_ = 6.60, ^∗∗^*p* < 0.01 vs. rest of the groups]; 40–50 μm [*F*_(__5_,_664__)_ = 8.42, ^∗∗^*p* < 0.01 vs. rest of the groups]; 50–60 μm [*F*_(__5_,_674__)_ = 6.51, ^∗∗^*p* < 0.01 vs. rest of the groups]) (Figure 2E). Neuron curvature ratio was also impaired after Col lesions and EPO treatment significantly ameliorated this situation both at P14 ([*F*_(__5_,_813__)_ = 5.78, ^∗∗^*p* < 0.01 vs. rest of the groups]) and P70 ([*F*_(__5_,_565__)_ = 5.901, ^∗∗^*p* < 0.01 vs. rest of the groups]) (Figure 2F). Spine density (number of spines/10 μm) was also restored after EPO treatment in the short (P14 [*F*_(__5_,_3985__)_ = 27.55, ^∗∗^*p* < 0.01 vs. rest of the groups, ‡‡*p* < 0.01 vs. Control, Control + EPO10 and Control + EPO20, ##*p* < 0.01 vs. Control and Control + EPO10]) and the long term (P70 [*F*_(__5_,_1091__)_ = 15.44, ^∗∗^*p* < 0.01 vs. rest of the groups, ‡‡*p* < 0.01 vs. Control, Control + EPO10 and Control + EPO20, –*p* < 0.01 vs. Control + EPO10]) (Figures 2G,H).

### EPO Treatment Reduces Central Bleeding in a GM-IVH Model

**FIGURE 3:**
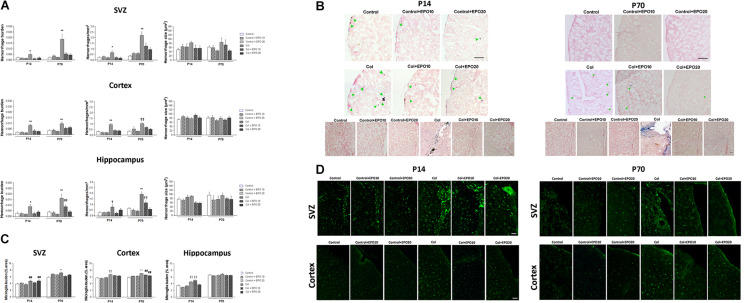
EPO treatment reduces vascular damage and inflammation after inducing a GM-IVH. **(A)** Hemorrhage burden was significantly increased in the SVZ after Col lesions and EPO10 and EP20 treatments reversed this effect, both in the short (P14) and the long term (P70) (P14: *p = 0.013 vs. rest of the groups and P70: **p < 0.01 vs. rest of the groups). The number of individual hemorrhages was reduced after EPO treatment (P14: *p = 0.021 vs. rest of the groups; P70: **p < 0.01 vs. rest of the groups), while individual hemorrhage size was not affected (P14: *p* = 0.169]; P70: *p* = 0.440]. When we analyzed the cortex we observed a similar profile and increased hemorrhage burden, after Col lesions, was reduced by EPO treatment (P14: **p < 0.01 vs. rest of the groups; P70: **p < 0.01 vs. rest of the groups), due to a reduction in the number of individual hemorrhages after the treatment (P14: **p < 0.01 vs. rest of the groups; P70: ††*p* < 0.01 vs. Control, Control + EPO10, Control + EPO20 and Col + EPO20), while hemorrhage size was not affected (P14: *p* = 0.123; P70: *p* = 0.296). In the hippocampus, increased hemorrhage burden in the ipsilateral side was reversed by EPO administration (P14: *p = 0.02 vs. rest of the groups; P70: **p < 0.01 vs. rest of the groups, ##*p* < 0.01 vs. Control + EPO10 and Control + EPO20). The number of individual hemorrhages was reduced after the treatment (P14: †*p* = 0.046 vs. Control, Control + EPO10, Control + EPO20 and Col + EPO10; and P70: **p < 0.01 vs. rest of the groups, ††*p* < 0.01 vs. Control, Control + EPO10, Control + EPO20, Col + EPO20) while hemorrhage size was not affected (P14: *p* = 0.005, no further differences detected; P70: *p* = 0.270). **(B)** Illustrative images of cortical Prussian blue staining and Congo red counterstain for hemorrhages. Increased bleeding after Col lesions was reduced by EPO treatment both at P14 and P70. Green arrow point at individual cortical hemorrhages. Scale bar = 250 μm. Bottom panels show increased hemorrhage burden in the SVZ from Col-lesioned animals, both at P14 and P70. Scale bar = 25 μm. **(C)** Microglia activation after Col lesions was reduced by EPO treatment at P14 and P70 in the SVZ (P14: ##*p* = 0.003 vs. Control and Control + EPO20; P70: **p < 0.01 vs. rest of the groups), cortex (P14: ‡‡*p* < 0.01; P70: **p < 0.01 vs. rest of the groups; ##*p* < 0.01 vs. Control) and hippocampus (P14: ‡‡*p* < 0.01 vs. Control, Control + EPO10 and Control + EPO20; P70: *p* = 0.098). **(D)** Illustrative example of microglia immunostaining for IBA-1 (green) in the SVZ and cortex from all groups under study. Increased microglia activation can be observed in Col-treated mice while EPO treatment significantly reduces this effect. Scale bar = 50 μm.

Increased hemorrhage burden in the SVZ after Col lesions was completely reversed by EPO10 and EPO20 administration at P14 [*F*_(__5_,_112__)_ = 11.089, ^∗^*p* = 0.013 vs. rest of the groups] and P70 [*F*_(__5_,_125__)_ = 11.089, ^∗∗^*p* < 0.01 vs. rest of the groups], due to a reduction in the number of individual hemorrhages (P14: [*F*_(__5_,_111__)_ = 2,76, ^∗^*p* = 0.021 vs. rest of the groups]; P70: [*F*_(__5_,_127__)_ = 15.36, ^∗∗^*p* < 0.01 vs. rest of the groups], while individual hemorrhage size was not affected (P14: [*F*_(__5_,_1111__)_ = 1.55, *p* = 0.169] P70 [*F*_(__5_,_149__)_ = 0.967, *p* = 0.440]) (Figure 3A). A similar profile was observed in the cortex and increased hemorrhage burden was reduced by EPO (P14 [*F*_(__5_,_271_) = 12.16, ^∗∗^*p* < 0.01 vs. rest of the groups]; P70 [*F*_(__5_,_277__)_ = 6.64, ^∗∗^*p* < 0.01 vs. rest of the groups]), number of individual hemorrhages was reduced by EPO (P14 [*F*_(__5_,_266__)_ = 20.75, ^∗∗^*p* < 0.01 vs. rest of the groups]; P70 [*F*_(__5_,_228__)_ = 10.01, ††*p* < 0.01 vs. Control, Control + EPO10, Control + EPO20 and Col + EPO20]) while hemorrhage size was not affected (P14 [*F*_(__5_,_553__)_ = 1.74, *p* = 0.123]; P70 [*F*_(__5_,_690__)_ = 1.22, *p* = 0.296]) (Figures 3A,B). The hippocampus showed a similar trend (hemorrhage burden: P14 [*F*_(__5_,_124__)_ = 2.79, ^∗^*p* = 0.02 vs. rest of the groups]; P70 [*F*_(__5_,_103__)_ = 13.01 ^∗∗^*p* < 0.01 vs. rest of the groups, ##*p* < 0.01 vs. Control + EPO10 and Control + EPO20]; number of hemorrhages: P14 [*F*_(__5_,_124__)_ = 2.33, †*p* = 0.046 vs. Control, Control + EPO10, Control + EPO20 and Col + EPO10] and P70 [*F*_(__5_,_116__)_ = 15.80, ^∗∗^*p* < 0.01 vs. rest of the groups, ††*p* < 0.01 vs. Control, Control + EPO10, Control + EPO20, Col + EPO20] and hemorrhage size: P14 [*F*_(__5_,_285__)_ = 3.42, *p* = 0.005, no further differences detected], P70 [*F*_(__5_,_286__)_ = 1.28, p = 0.270]).

### Inflammation Is Reduced in a GM-IVH After EPO Treatment

After Col lesions, microglia burden was analyzed in the SVZ, cortex and hippocampus at P14 and P70. Col-induced GM-IVH increased microglia burden, and EPO treatment limited this effect when we analyzed the SVZ at P14 [*F*_(__5_,_817__)_ = 3.63, ##*p* = 0.003 vs. Control and Control + EPO20] and at P70 [*F*_(__5_,_768__)_ = 4.71, ^∗∗^*p* < 0,01 vs. rest of the groups] (Figures 3C,D). We observed a similar profile when we analyzed the cortex, and EPO treatment reduced microglia burden at P14 ([*F*_(__5_,_3870__)_ = 12.66, ‡‡*p* < 0,01 vs. Control, Control + EPO10 and Control + EPO20]) and P70 ([*F*_(__5_,_3521__)_ = 18.122, ^∗∗^*p* < 0,01 vs. rest of the groups; ##*p* < 0.01 vs. Control]) (Figures 3C,D). In the hippocampus, EPO treatment successfully reduced microglia burden at the highest dose (EPO20) at P14 [*F*_(__5_,_775__)_ = 14.78, ‡‡*p* < 0.01 vs. Control, Control + EPO10 and Control + EPO20], whereas differences did not reach statistical significance at P70 [*F*_(__5_,_687__)_ = 1.86, *p* = 0.098]) (Figure 3C).

### EPO Treatment Reduces Tau Phosphorylation in a GM-IVH Murine Model

**FIGURE 4:**
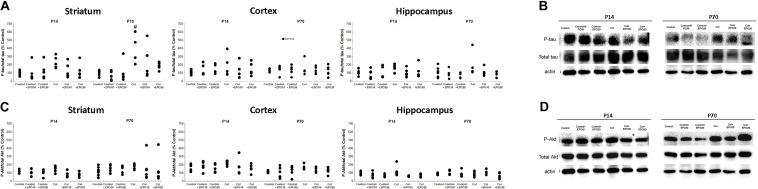
EPO treatment reduces tau and Akt phosphorylation after Col lesions. **(A)** Increased tau phosphorylation after Col lesions was reduced after EPO treatment at P14 although differences did not reach statistical significance (striatum: *p* = 0.193; cortex: *p* = 0.922 and hippocampus: *p* = 0.504]). A similar profile was observed when we analyzed animals in the long term (P70) (striatum: #*p* = 0.023 vs Control + EPO20; cortex: *p* = 0.658 and hippocampus: *p* = 0.522). **(B)** Illustrative example of phospho-tau/total tau striatum westerns blots at P14 and P70, showing an increased phosphorylation after Col lesions at P70, that is reduced by EPO treatment. **(C)** Increased phosphor-Akt/total Akt ratios after Col lesions were reduced by EPO treatment in the ipsilateral hemisphere although differences did not reach statistical significance at P14 (striatum: *p* = 0.127; cortex: *p* = 0.552; hippocampus: *p* = 0.055) or at P70 (striatum: *p* = 0.931; cortex: *p* = 0.425; hippocampus: *p* = 0.475). **(D)** Illustrative example of striatum phosphor-Akt/total Akt western blots at P14 and P70, after EPO treatments.

Increased tau phosphorylation after Col lesions was reduced by EPO treatment at P14, however, differences did not reach statistical significance (striatum [*F*_(__5_,_25__)_ = 1.61, *p* = 0.193]; cortex: [*F*_(__5_,_25__)_ = 0.275, *p* = 0.922] and hippocampus: [*F*_(__5_,_24__)_ = 0.8889, *p* = 0.504]) (Figures 4A,B). At P70 EPO limited tau hyperphosphorylation in the striatum [*F*_(__5_,_26__)_ = 3.17, #*p* = 0.023 vs. Control + EPO20]) and while a similar profile was observed in the hippocampus, differences did not reach statistical significance [*F*_(__5_,_24__)_ = 0.860, *p* = 0.522]. No differences were observed in the cortex at P70 [*F*_(__5_,_24__)_ = 0.659, *p* = 0.658] (Figures 4A,B).

### Effect of EPO Treatment on Akt Phosphorylation in a GM-IVH Murine Model

We detected a slight increase in phosphoAkt/total Akt ratios after inducing a GM-IVH. EPO treatment reduced this effect in the ipsilateral hemisphere although no differences were observed at P14 (striatum [*F*_(__5_,_25__)_ = 1.91, *p* = 0.127]; cortex [*F*_(__5_,_25__)_ = 0,813, *p* = 0.552], hippocampus [*F*_(__5_,_24__)_ = 2.54, *p* = 0.055]) (Figures 4C,D) or P70 (striatum P70 [*F*_(__5_,_30__)_ = 0.260, *p* = 0.931]; cortex [*F*_(__5_,_22__)_ = 1.029, *p* = 0.425]; hippocampus [*F*_(__5_,_31__)_ = 0.930, *p* = 0.475]) (Figures 4C,D).

### EPO Treatment Reduces Alterations in Plasma Markers After Col Lesions

**FIGURE 5:**
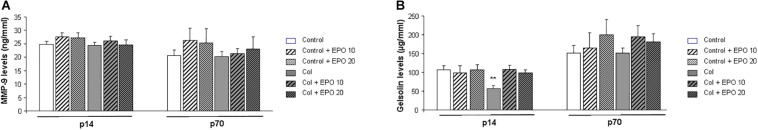
Effect of EPO treatment on peripheral markers of GM-IVH. **(A)** EPO treatment does not affect MMP9 plasma levels at P14 (*p* = 0.633) or P70 (*p* = 0.453). **(B)** EPO treatment restores plasma gelsolin levels after inducing a GM-IVH in the short term, while differences are no longer detected in the long term (P70) (P14 **p = 0.002 vs. rest of the groups, P70 *p* = 0.575).

MMP9 plasma levels were not significantly affected (P14 [*F*_(__5_,_63__)_ = 0.689, *p* = 0.633]; P70 [*F*_(__5_,_63__)_ = 0.953, *p* = 0.453]) (Figure 5A). On the other hand, gelsolin levels were reduced after Col lesions at P14, as described (Segado-Arenas et al., 2018), while EPO treatment counterbalanced this situation [*F*_(__5_,_60__)_ = 4.372, ^∗∗^*p* = 0.002 vs. rest of the groups]. Differences were no longer significant in the long term (P70 [*F*_(__5_,_60__)_ = 0.770, *p* = 0.575]) (Figure 5B).

## Discussion

Germinal matrix-intraventricular hemorrhage remains one of the most serious complications of the PTI (Mukerji et al., 2015) and has no successful treatment (Brouwer et al., 2014). Most of the current approaches have focused on strengthening the GM or stabilizing brain blood flow to prevent the GM-IVH (Ballabh, 2010). Prenatal glucocorticoids (Carson et al., 2016), indomethacin (Fowlie and Davis, 2003) or phenobarbital have been proposed as feasible treatments. On the other hand, it has also been suggested that stem cells therapy might provide an encouraging approach. Nevertheless, some limitations still exist, such as choosing the right cells, the appropriate patients or routes of administration. Also, safety issues remain to be solved and significantly limit the use of stem cells in the clinic (Chang et al., 2017).

Recombinant human EPO is regularly used to prevent or treat anemia of the PTI and studies in patients reveal that the early use of erythropoiesis-stimulating agents may reduce the incidence of GM-IVH and be neuroprotective (Neubauer et al., 2010). The doses of EPO used in our study are high when compared with those used in PTI patients (Juul et al., 2020). Dose conversion remains a relevant issue in experimental animal models (for review Nair and Jacob, 2016) and doses tend to be reduced as we ascend in the phylogenetic scale. The doses used in our study are in the range of those previously used in other neonate murine models (Kaindl et al., 2008; Fan et al., 2011; Hoeber et al., 2016; Zhang et al., 2020). Moreover, safety of EPO has been previously addressed with large doses of EPO, even in extremely low weight infants (McAdams et al., 2013). Erythropoiesis-stimulating agents may also have a positive effect on neurodevelopmental impairment of the PTI (for review see Ohlsson and Aher, 2017, 2020). Following this idea, and while results remain controversial, EPO may specifically provide beneficial effects on the neurodevelopment outcome in the PTI without severe adverse side effects (Wang et al., 2015). Nevertheless later updates on the beneficial effects of EPO remain controversial and the promising, but conflicting, results related to EPO as a neuroprotective agent require further study (Ohlsson and Aher, 2020). Moreover, several clinical trials have been in progress in the last few years assessing EPO in neonatal populations (Maxwell et al., 2017). The multicenter (19 sites, 30 hospitals), randomized, double-blind trial of EPO, PENUT (Preterm Epo Neuroprotection), has recently shown limited effects on very PTI after ≈2 years follow up. The PENUT study is an extremely relevant study on the field, however, individual groups were not analyzed and it remains possible that cognitive and physical problems might become evident later in life (Juul et al., 2020). Following this idea, Volpe (2020) has commented this aspect as a main limitation, also acknowledged in the study, since cognitive testing at 2 years of age is not as reliable as testing at later ages. In this sense, other studies comparing early assessments (before 3 years of age) with later cognitive tests at school age, showed a relatively low pooled sensitivity (55%) of early assessments to identify school-age cognitive deficits. Moreover, EPO is especially successful in protecting preoligodendrocytes. White matter development is an active process that continues well after infancy and it’s closely related to specific cognitive functions that may be affected at later stages. Therefore Volpe suggests the necessity of testing longer duration of treatment, reaching full-term equivalent age or even longer (Volpe, 2020). A previous meta-analysis has also showed that that prophylactic EPO improves cognitive development (Fischer et al., 2017) and early EPO treatment may decrease the rates of necrotizing enterocolitis as well as brain complications, including IVH and periventricular leukomalacia (Ohlsson and Aher, 2017). Moreover, it seems that an early high dose of EPO has a weak but widespread effect on brain structural connectivity network in very PTI, supporting a trophic effect of EPO that increases network segregation, predominantly in local connections (Jakab et al., 2019). Nevertheless, recent updates report a limited success of EPO treatment (Ohlsson and Aher, 2020), supporting further studies given the controversial outcomes. Altogether, and given the present results, EPO administration is not currently recommended because the benefits are not fully established (Ohlsson and Aher, 2017; Fleiss and Gressens, 2019) and the underlying mechanisms and specific effects in the GM-IVH have not been studied in depth. At this point, as it has previously been stressed out there are no preclinical data showing the neuroprotective effects of EPO in a model of encephalopathy of prematurity, limiting the understanding of the best paradigm to deliver neuroprotection in this population (Fleiss and Gressens, 2019).

In order to help disentangle the effects of EPO treatment, we have treated with EPO a recently characterized murine model of GM-IVH of the preterm newborn, induced by Col administration (Segado-Arenas et al., 2018). While evident limitations persist, it is noteworthy that this model reproduces morphological, functional and neurodevelopmental alterations of the GM-IVH. Col administration in the lateral ventricle breaks the extracellular matrix that surrounds the GM capillaries, increases blood brain barrier permeability and induces bleeding (Segado-Arenas et al., 2018). Moreover Col administration to neonate (P7) CD1 mice results in brain atrophy, ventricle enlargement, increased inflammation, widespread small vessel damage and cognitive impairment that are still detected in the adulthood (Segado-Arenas et al., 2018), as observed in the clinic. EPO treatment, at 10.000 or 20.000 IU/Kg significantly ameliorated learning and memory alterations in the long term (P70). Although the fact that young P14 mice cannot be assessed at cognitive level is limiting, early histopathological complications at P14 match the functional outcomes at P70. In our hands, motor-related alterations are limited with this approach, and we cannot exclude that larger lesions could reproduce motor impairment as regularly observed in the clinic (Hinojosa-Rodríguez et al., 2017). While the studies on the effect of EPO in neonates with GM-IVH are limited (Ruegger et al., 2015), our data are in accordance with a seminal study by Neubauer et al. (2010) in which children with GM-IVH showed a remarkably strong benefit from EPO treatment at 5 years of age and later. While some controversy persists (Leuchter et al., 2014; Natalucci et al., 2016), these observations are also in line with studies showing that EPO improves cognitive outcomes in PTI both early in life (18–22 months) (Wang et al., 2015) and once the patients reach school (3.5–4 years old) (Ohls et al., 2016).

Morphopathological changes after Col lesions resemble those detected in PTI with GM-IVH, including complicated post-hemorrhagic ventricular dilatation (Brouwer et al., 2014). Moreover, these changes are directly associated with an overall brain atrophy and bad prognosis (Saliba et al., 1990). Also, the neuronal population is compromised around the ventricles (Georgiadis et al., 2008), as well as in areas located far from the lesion area, such as the cortex, supporting an overspread long-lasting damage, as previously described (Segado-Arenas et al., 2018). The mechanisms implicated have not been elucidated, and atrophy has been attributed to both loss of ischemic infracted tissue and defective development of the damaged areas (Semple et al., 2013). In our hands, curvature ratio, as an indicator of neuron wellness (Infante-Garcia et al., 2015), neuronal complexity and spine density were compromised in mice after GM-IVH, while EPO treatment rescued these alterations, supporting its neurotrophic role (Juul and Pet, 2015) and neuroprotective effect at different levels. To our knowledge, the role of EPO in a model of GM-IVH of the PTI has not been previously assessed, however, in line with our observations, EPO protects against striatum atrophy, hippocampus injury, and white matter loss in a model of hypoxia-ischemia in neonatal mice (Fan et al., 2011).

We observed an increase of widespread hemorrhages after Col lesions, as previously described (Segado-Arenas et al., 2018). Hemorrhage density and burden seem to be higher in the SVZ at later ages (P70), suggesting that small vessel damage might be a long-term consequence of the GM-IVH. We never addressed age differences, since P14 brains are extremely immature (Semple et al., 2013) and can hardly be compared to fully developed P70 brains. The fact that EPO treatment successfully counterbalances this effect might be attributed to its role in erythropoiesis, that requires iron utilization, and therefore reduces the potential toxicity of free iron (Juul and Ferriero, 2014). Likewise, EPO promotes revascularization in hypoxic-ischemic neonatal models (Iwai et al., 2007). Previous studies have also shown that EPO induces angiogenesis through the production of vascular endothelial growth factor, protecting capillaries (Wang et al., 2008) and bood brain barrier integrity (Marti et al., 2000). Col lesions also provoked an inflammatory response that is commonly observed in PTI (Juul and Ferriero, 2014). The antioxidant and anti-inflammatory effects of EPO have been widely assessed in different models of brain insult (Wei et al., 2017) and in our hands EPO treatment significantly reduces microglia activation after GM-IVH induction. Iba-1 immunostatining is classically used to label microglia, however, we only analyzed microglia burden and it is possible that more complex inflammatory changes are not covered with our approach. Previous studies have shown that microglia express EPO receptors (Nagai et al., 2001) and that EPO may reduce motility, as an important feature of microglial pathological reaction to damage (Mitkovski et al., 2015), while promoting the polarization of microglia toward the protective M2 phenotype (Wei et al., 2017).

Hyperphosphorylated tau is a toxic pathological hallmark of neurodegenerative disorders, increased in this and other models with central hemorrhages (Infante-Garcia et al., 2016; Segado-Arenas et al., 2018). Col lesions increased tau phosphorylation in the striatum, as the closest area to the ventricle. EPO slightly reduced this situation in the striatum and no effect was detected in the cortex or the hippocampus. Previous studies have reported that EPO, alone or combined with other treatments, may reduce tau phosphorylation associated to different cognitive disorders (Kang et al., 2010; Vinothkumar et al., 2019). Even though glycogen synthase kinase-3β inhibition reduces tau phosphorylation (Maqbool et al., 2016), enhances myelination, improves clinical recovery in a model of GM-IVH (Dohare et al., 2018a) and restores altered neurogenesis in preterm patients with intraventricular hemorrhage (Dohare et al., 2018b), the narrow effect in our studies limits further conclusions at this level. Also, since Akt is implicated in cell cycle regulation, cell survival and apoptosis, it is feasible that the beneficial effects observed after EPO treatment could be Akt-mediated. Previous studies have shown that Akt phosphorylation is increased in subarachnoid hemorrhage models (Guo et al., 2018). It has also been pointed out that EPO, in combination with insulin-like growth factor-1, may contribute to increase Akt activation (Kang et al., 2010). However, we only observed a slight increase in phopho-Akt levels after inducing a GM-IVH.

MMP9 levels were not affected, in agreement with previous studies (Segado-Arenas et al., 2018). On the other hand, plasma gelsolin levels were significantly reduced in mice after GM-IVH, as previously reported (Segado-Arenas et al., 2018). Plasma gelsolin is a feasible peripheral marker of central complications and low plasma gelsolin levels have been associated with poor outcomes in adult brain hemorrhagic alterations (Chou et al., 2011), in premature infants (Kose et al., 2014) and other newborn complications (Benavente-Fernandez et al., 2018). EPO treatment significantly restores plasma gelsolin levels, suggesting that its positive effects at central level are also detected in the periphery and reinforcing further studies on the utility of plasma gelsolin as a prognostic tool.

## Conclusion

Erythropoietin is a safe, already approved drug to treat other complications of the PTI. Altogether, our data support further assessment of EPO (Volpe, 2020) as a feasible treatment to protect against central complications associated to GM-IVH in the short (P14) and the long term (P70). Moreover we provide evidence of gelsolin, as a feasible marker for a devastating disease. Ultimately, our study helps to further understand the pathophysiology underlying the IVH of the preterm newborn and the best paradigms to define new protection for PTI (Fleiss and Gressens, 2019).

## Data Availability Statement

The raw data supporting the conclusions of this article will be made available by the authors, without undue reservation upon reasonable request.

## Ethics Statement

The animal study was reviewed and approved by Junta de Andalucia (Guidelines for Care and Use of Experimental Animals, European Commission Directive 2010/63/UE and Spanish Royal Decree 53/2013) and the University of Cádiz Bioethics Committee.

## Author Contributions

CH-B, CI-G, DS-S, and AM: experiments design, data acquisition, analysis, and interpretation. AC-R, CM-G, CL-P, and MB-M: data acquisition and analysis. IB-F: study concept and design and critical revision of manuscript for intellectual content. SL-L and MG-A: study concept and design, drafting, and critical revision of manuscript for intellectual content. All authors read and approved the final manuscript.

## Conflict of Interest

The authors declare that the research was conducted in the absence of any commercial or financial relationships that could be construed as a potential conflict of interest.
